# A Magnetic Bead-Based Method for Concentrating DNA from Human Urine for Downstream Detection

**DOI:** 10.1371/journal.pone.0068369

**Published:** 2013-07-08

**Authors:** Hali Bordelon, Patricia K. Russ, David W. Wright, Frederick R. Haselton

**Affiliations:** 1 Department of Biomedical Engineering, Vanderbilt University, Nashville, Tennessee, United States of America; 2 Department of Chemistry, Vanderbilt University, Nashville, Tennessee, United States of America; Naval Research Laboratory, United States of America

## Abstract

Due to the presence of PCR inhibitors, PCR cannot be used directly on most clinical samples, including human urine, without pre-treatment. A magnetic bead-based strategy is one potential method to collect biomarkers from urine samples and separate the biomarkers from PCR inhibitors. In this report, a 1 mL urine sample was mixed within the bulb of a transfer pipette containing lyophilized nucleic acid-silica adsorption buffer and silica-coated magnetic beads. After mixing, the sample was transferred from the pipette bulb to a small diameter tube, and captured biomarkers were concentrated using magnetic entrainment of beads through pre-arrayed wash solutions separated by small air gaps. Feasibility was tested using synthetic segments of the 140 bp tuberculosis IS6110 DNA sequence spiked into pooled human urine samples. DNA recovery was evaluated by qPCR. Despite the presence of spiked DNA, no DNA was detectable in unextracted urine samples, presumably due to the presence of PCR inhibitors. However, following extraction with the magnetic bead-based method, we found that ∼50% of spiked TB DNA was recovered from human urine containing roughly 5×10^3^ to 5×10^8^ copies of IS6110 DNA. In addition, the DNA was concentrated approximately ten-fold into water. The final concentration of DNA in the eluate was 5×10^6^, 14×10^6^, and 8×10^6^ copies/µL for 1, 3, and 5 mL urine samples, respectively. Lyophilized and freshly prepared reagents within the transfer pipette produced similar results, suggesting that long-term storage without refrigeration is possible. DNA recovery increased with the length of the spiked DNA segments from 10±0.9% for a 75 bp DNA sequence to 42±4% for a 100 bp segment and 58±9% for a 140 bp segment. The estimated LOD was 77 copies of DNA/µL of urine. The strategy presented here provides a simple means to achieve high nucleic acid recovery from easily obtained urine samples, which does not contain inhibitors of PCR.

## Introduction

Relative to many other types of patient samples, urine samples are easy to obtain. Therefore, they are frequently analyzed for biomarkers of disease. Urine biomarkers include sugars for the diagnosis of diabetes mellitus, proteins for the diagnosis of liver and kidney disorders, and bacteria for the diagnosis of urinary tract infections [1]. In addition, transrenal DNA (Tr-DNA) sequences have been shown to be applicable in pathogen detection. As human cells and microorganisms break down within the body, small nucleic acid fragments are thought to circulate in the blood stream, and subsequently pass through the kidneys and into the urine [2], [3]. In healthy individuals, these Tr-DNA fragments have been shown to be less than 250 bp in length [4]. Tr-DNA sequences specific to *Mycobacterium tuberculosis* [5]–[7], urinary tract infections [8], *Plasmodium* [9], and *Schistosoma mansoni* [10] have been recovered from urine, pointing to Tr-DNA as a promising new biomarker for urinalysis.

The noninvasive collection of urine samples is advantageous in low resource settings where lack of automated equipment and skilled technicians, cultural taboos, or patient age make more invasive sample collection difficult [11]. Compared to blood, sputum, or cheek swab samples, urine sample volume is large, typically 10 to 100 mL, which maximizes the availability of the biomarker of interest. This is a distinct advantage when detecting a biomarker that is expected to be present at relatively low concentrations, like Tr-DNA. However, urine contains inhibitors that interfere with downstream nucleic acid-based detection strategies such as PCR [11], [12]. Additionally, isothermal PCR and other nucleic acid-based assays being developed for use in low resource settings are frequently tested using commercial nucleic acid extraction kits that may be inappropriate for use in these settings. Thus, the need for nucleic acid extraction and concentration is a well-recognized roadblock to performing nucleic acid-based detection in low resource settings [11], [13], [14].

In order for PCR or other nucleic acid-based assays to detect DNA in urine samples, the DNA must be separated from inhibitors contained in the sample. Current methods to extract nucleic acids for nucleic acid-based assays are either not amenable to a large volume patient sample such as urine, are not appropriate to a low resource setting, or both. Commercially-available extraction kits for use in laboratories, such as Exgene Clinic SV (Biomol, GmbH), Norgen Urine DNA Isolation kit (Norgen Biotek Corporation), and QIAmp DNA kit (Qiagen), utilize spin columns to selectively adsorb nucleic acids and remove potential inhibitors to downstream assays. However, these columns require relatively expensive equipment (e.g. centrifuge), disposable laboratory supplies, and trained personnel. Products such as InstaGene Matrix (Bio-Rad) also remove PCR inhibitors from patient samples but dilute the DNA instead of concentrating it. Self-contained microfluidic devices embedded with silica beads or posts have been developed for one-step nucleic acid extractions [15]. However, the small diameter of microfluidic channels restricts the silica surface area available for biomarker adsorption as well as the maximum sample volume that can be flowed through the channels (typically <500 µL) [14]. This limits the utility of microfluidic devices for processing large volume samples like urine. We report here a means of extracting and concentrating DNA from up to 5 mL urine samples without pipetting and centrifugation steps necessary in commercial DNA extraction kits.

Magnetic beads are an effective tool for extracting and concentrating biomarkers. Using this dispersed solid phase approach, magnetic beads are mixed with large volumes of patient sample to capture biomarkers. The magnetic beads are then recollected magnetically, and the captured biomarkers are released from the bead surface into a more amenable reaction buffer. This has led to their use in a number of bioassay applications [16]–[18]. The coating on the magnetic bead surface is one of the key factors to this approach. The adsorption of nucleic acids to silica in the presence of high concentrations of chaotropic salts is used extensively to extract nucleic acids from complex sample matrices into inhibitor-free solutions in bead-based methods as well as in a number of other formats. Additionally, the mobility of magnetic beads allows transfer of adsorbed nucleic acids into smaller volumes and, therefore, leads to more concentrated solutions [19].

The ideal nucleic acid extraction technology for a low resource setting would require minimal processing steps, enable the use of large sample volumes to maximize biomarker recovery, and be self-contained to limit operator exposure to samples during processing 14,20. We have previously reported the development of a low resource “extraction cassette” for the purification of RNA [21] and protein [22] from small volume patient samples. The extractions were performed in a single length of small diameter tubing pre-arrayed with processing solutions separated from one another by air gaps or mineral oil held in place by surface tension. After biomarker capture on the bead surface, the magnetic beads were pipetted into the small diameter tubing, and an external permanent magnet was pulled along the length of the tubing to wash and release biomarkers into a final buffer for subsequent testing.

In these prior assays, biomarker capture by magnetic beads was performed in a multi-step process using refrigerated reagents added to a centrifuge tube. In this report, we eliminate the need for multiple pipetting steps, refrigerated reagents, and the precise volume transfer to the small diameter tubing. In addition, we have modified the magnetic bead and tubing components of our “extraction cassette” design based on the optimization studies reported by Adams et. al. [23]. The end result is a simple means to collect DNA biomarkers present in large volume urine samples in a transfer pipette bulb and to concentrate the biomarkers into a small volume using our preloaded extraction cassette for subsequent nucleic acid-based detection.

## Materials and Methods

### Preparation of Pooled Human Urine Samples

Twenty disease-negative urine samples were obtained from the Vanderbilt University Molecular Infectious Disease Laboratory as “on-the-shelf” samples that contained no patient information. An IRB exemption was granted from the Vanderbilt University Institutional Review Board for use of these samples. There were two ways to use the human urine samples. They could either be individually spiked prior to extracting, or they could be pooled and aliquotted prior to spiking. In these studies, the urine samples were pooled allowing us to minimize variability in the concentration of PCR inhibitors that would be expected between individual urine samples. Fifteen milliliters of each sample were pooled, pipetted into 1 mL aliquots, and stored at −80°C. Immediately prior to use, the samples were thawed at room temperature and spiked, in most cases, with a 140 bp synthetic DNA target from the IS6110 sequence of *M. tuberculosis* (Integrated DNA technologies, Coralville, Iowa). The one exception is the sequence length studies described below.

### Quantitative PCR

A 129 bp amplicon of the IS6110 sequence was amplified using forward primer 5′-ACCAGCACCTAACCGGCTGTGG-3′ and reverse primer 5′-CATCGTGGAAGCGACCCGCCAG-3′ [7]. Amplification reactions were performed in a 25 µL volume using 5 µL of DNA template and the Qiagen QuantiTect SYBR Green PCR kit (Qiagen, Germantown, MD) according to manufacturer’s instructions. Thermal cycling consisted of 95°C for 15 minutes to activate the Taq DNA polymerase and 40 cycles of 95°C for 15 s, 62°C for 30 s, and 72°C for 30 s using a Rotor-Gene Q thermal cycler (Qiagen, Germantown, MD). Product specificity was confirmed using melting curve analysis. Data were collected and C_t_ values recorded by Rotor-Gene Q software and converted to number of DNA copies per µL using a standard curve. The primer efficiency for each standard curve was also determined using the Rotor-Gene Q software.

### Preparation of Urine Collection Pipettes

The urine collection pipettes were prepared by drawing 1 mL of DNA-silica adsorption buffer (4 M guanidine thiocyanate, 25 mM sodium citrate, pH 7.0) containing 6×10^8^ Dynabeads® MyOne™ Silane magnetic beads (Life Technologies, Grand Island, NY) into the bulb of a 5 mL, fine tipped transfer pipette (Samco Scientific, San Fernando, CA, cat. # 232–20S). The contents of the transfer pipettes were frozen for 2 hours at −80°C, after which the pipettes were transferred to a Labconco bulk tray dryer (Labconco, Kansas City, MO) and lyophilized for ∼18 hours. Following lyophilization, the transfer pipettes were stored at room temperature for up to twelve weeks until use.

### DNA Extraction from Pooled Human Urine Samples

**Figure 1:**
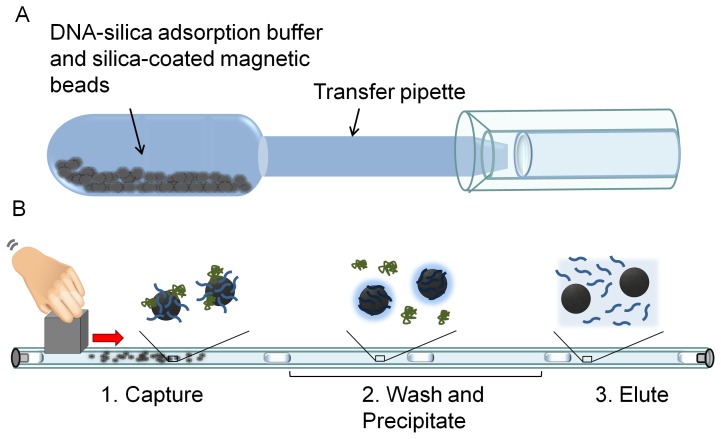
The two components of the magnetic bead-based extraction method. A) DNA is adsorbed to silica-coated magnetic beads previously lyophilized with DNA-silica adsorption buffer in the bulb of a transfer pipette. After mixing, the beads are transferred directly into the small diameter tubing by depressing the bulb. B) The small diameter tubing “extraction cassette” is shown with processing solutions separated by air gaps. DNA adsorbed to silica-coated magnetic beads is pulled through each processing solution by an external magnet and eluted in the final water chamber.

The two components of this magnetic bead-based approach are shown in **Figure 1**. Based on the optimization studies performed by Adams et. al. the previously described “extraction cassette” was modified by using fluorinated ethylene propylene (FEP) tubing in place of Tygon® R-3603 tubing [23]. The tubing was prepared by preloading a 60 cm length of 1.6 mm inner diameter (i.d.) FEP tubing (Saint-Gobain Performance Plastics, Akron, OH) with DNA precipitation buffer (300 µL of 80% ethanol, 5 mM potassium phosphate, pH 8.5), DNA wash solution (300 µL of 70% ethanol), and DNA eluent (50 µL of molecular grade water). Each solution was pipetted sequentially into the tubing and separated from the next by a 4 µL air gap (2 mm in length). A 1 mL human urine sample was spiked with 5 µL of IS6110 DNA to a concentration of 5×10^5^ copies/µL. Prior to extraction, the samples were drawn into the bulb of the transfer pipette, which was shaken vigorously by hand for 30 s to dissolve the lyophilized salts, then gently for 30 s to allow DNA adsorption to the silica surfaces of the beads. After mixing, the transfer pipette was squeezed to transfer the sample from the bulb to the tip of the pipette. The end of the pipette was then inserted into the preloaded small diameter tubing and the sample was transferred into the tubing (**Figure 1A**). Stainless steel #1 machine screws (McMaster Carr Supply Co, Elmhurst, IL, cat. # 90065A054) were threaded into both ends of the FEP tubing to establish an airtight seal. A 2.5 cm cube-shaped neodymium magnet (Emovendo, Petersburg, WV) adjacent to the DNA-silica adsorption chamber was used to collect the beads. Although one bead is not visible to the naked eye, the quantity of beads used here (6×10^8^ beads) is readily perceived as a dark brown clump. The beads were pulled through the air gaps and each successive solution at ∼4 mm/s (**Figure 1B**). Beads were dispersed in the DNA precipitation buffer and DNA wash solution by rapidly moving the magnet back and forth along the chamber. Beads were then collected and pulled into the next solution. Finally, the beads were dispersed throughout the eluent for ∼10 s, collected, and pulled back into the DNA wash chamber. Each DNA extraction was completed in ∼15 minutes.

DNA recovery was quantified by PCR. To prepare the extracted sample for PCR, the elution chamber was excised from the small diameter tubing using a razor blade, and the eluate was collected by holding the tubing over a 500 µL centrifuge tube. Additional extractions were performed using the commercially available Norgen Biotek urine extraction mini kit (Thorold, Ontario, Canada), performed according to manufacturer’s instructions on a 1 mL urine sample. PCR was also performed on 5 µL of an unextracted urine sample containing 5×10^5^ copies/µL of IS6110 DNA. DNA recovery was calculated by dividing the total number of copies extracted by the initial number of copies present in the sample and multiplying by 100%.

### Effect of Urine Sample Volume on Final DNA Concentration in the Eluate

The recovery of spiked DNA from 1, 3 and 5 mL urine samples, each containing 5×10^5^ copies/µL of spiked DNA, was determined following magnetic bead-based extraction. Each sample was drawn into the bulb of the lyophilized urine collection pipette, which was shaken vigorously for 30 s to dissolve the lyophilized salts, then gently for 30 s to distribute the magnetic beads for nucleic acid adsorption. To avoid transferring volumes greater than 1 mL into the small diameter tubing and to limit the total length of the small diameter tubing to 60 cm, the magnetic beads were collected to one side of the pipette bulb using a permanent magnet, and all but 1 mL of each sample was transferred into a waste container for disposal. The remaining 1 mL urine sample containing all magnetic beads was then transferred into the small diameter tubing. Finally, the magnetic beads were processed using an external magnet, the eluate collected, and the DNA amplified and quantified by PCR as described above.

### Comparison of Urine Collection Pipette to Freshly Prepared DNA-silica Adsorption Buffer for DNA Capture

One milliliter pooled human urine samples were extracted as described above by capturing DNA onto silica-coated magnetic beads contained within the urine collection pipette, transferring the sample into the small diameter tubing, and processing the sample using an external magnet. The extractions were performed using urine collection pipettes containing lyophilized DNA-silica adsorption buffer and magnetic beads that had been stored at room temperature for 0, 4, 8, or 12 weeks prior to use. For comparison, the DNA-silica adsorption step was performed in a 2 mL centrifuge tube with freshly prepared DNA-silica adsorption buffer and magnetic beads. One milliliter of DNA-silica adsorption buffer and 6×10^8^ silica-coated magnetic beads were pipetted into the centrifuge tube followed by a 1 mL DNA-spiked pooled human urine sample. The contents of the tube were gently shaken for 60 seconds to allow mixing and DNA adsorption to the silica beads. Finally, the sample was transferred to the small diameter tubing by pipetting and processed as described above for DNA extraction. After processing, the recovery of IS6110 DNA from each sample was calculated following PCR.

### Effect of Spiked DNA Concentration of DNA Recovery

To determine the effect of spiked DNA concentration on DNA recovery from human urine samples, multiple concentrations of spiked DNA were extracted and analyzed by PCR. One milliliter human urine samples were spiked with 5 µL of TE buffer containing 0, 5×10^3^, 5×10^4^, 5×10^5^, 5×10^6^, or 5×10^8^ copies of TB IS6110 DNA. Following extraction the DNA was quantified by PCR.

### PCR Limit of Detection

The limit of detection was determined for the PCR protocol described above. A 1 mL urine sample containing no TB IS6110 DNA was extracted using the magnetic bead-based method. PCR was performed on the extracted sample, and the number of copies detected in the blank sample was determined by referencing a standard curve run in TE buffer. The PCR LOD was defined to be 3 s.d. above the average number of calculated copies from the extracted unspiked urine sample.

The minimum concentration of DNA that must be spiked into a human urine sample to be detectable in the eluate following extraction with the magnetic bead-based method, given the LOD of the PCR protocol used in this study, was determined. Five microliters of TE buffer containing 0, 5×10^3^, 5×10^4^, 5×10^5^, 5×10^6^, or 5×10^8^ copies of TB IS6110 DNA were added to each 1 mL urine sample. Following extraction with the magnetic bead-based method, the number of copies detected in the eluate of each extracted sample was determined by PCR. The approximate number of copies that must be spiked into the initial urine sample to be detectable in the eluate at the PCR LOD was determined as the point which intersects this PCR-concentration curve.

### Effect of DNA Length on DNA Adsorption to Silica

The effect of DNA target length on recovery of DNA using magnetic bead-based extraction was studied to determine the limitations of DNA adsorption to the silica-coated magnetic beads. Human urine samples (1 mL) were spiked with 5×10^8^ copies of DNA that was 75, 100, or 140 bp in length. The samples were extracted using the magnetic bead-based extraction, and the recovery of DNA for each sample was determined following PCR amplification by referencing a standard curve. A 67 bp fragment of each target was amplified using forward primer 5′-ACCAGCACCTAACCGGCTGTGG-3′ and reverse primer 5′-GTAGGCGAACCCTGCCCAGGTC-3′ [7], with cycling conditions identical to those described above.

### Adsorption and Elution Kinetics of Silica-coated Magnetic Beads

The optimum adsorption and elution times for DNA adsorption to and elution from silica-coated magnetic beads was determined by comparing the recovery of DNA for adsorption or elution times ranging from 30 to 210 s. For adsorption studies, 5×10^8^ copies of TB IS6110 DNA were spiked into 1 mL human urine samples and drawn into a transfer pipette bulb containing DNA-silica adsorption buffer and magnetic beads as described above. The pipette was shaken vigorously for 30 s to dissolve the salts, and then gently for an additional 0, 30, 60, or 180 seconds. For elution studies, 5×10^8^ copies of TB IS6110 DNA were extracted from 1 mL human urine samples using the magnetic bead-based extraction with an adsorption time of 30 s. After dispersal in the final elution chamber, the beads were incubated at room temperature for 30, 60, 90, or 210 seconds before being collected and removed from the chamber. Each sample was collected and IS6110 DNA was amplified by PCR. The extracted DNA was quantified by referencing a standard curve. The recovery of DNA was determined by dividing the total number of extracted DNA copies by the input number of DNA copies and multiplying by 100%.

### Effect of Number of Magnetic Beads on DNA Recovery

The recovery of spiked DNA from 1 mL urine samples was determined following magnetic bead-based extraction performed with different numbers of magnetic beads. For each extraction, a urine collection pipette was prepared with either 12×10^8^, 6×10^8^, 3×10^8^, or 1×10^8^ magnetic beads, and the magnetic bead-based extraction was performed on each spiked 1 mL urine sample as described above.

### DNA Extraction Capacity of the Silica-coated Magnetic Beads

The maximum mass of DNA that could be extracted with the silica-coated magnetic beads was determined by spiking 100 µL of TE buffer with increasing quantities of calf thymus DNA (Sigma Aldrich, St. Louis, MO) and quantifying the mass of DNA extracted. One hundred µL of TE buffer was spiked with 2.5, 5, 7, 10, or 20 µg of calf thymus DNA. Each sample was mixed with 300 µL of DNA-silica adsorption buffer and 6×10^8^ silica-coated magnetic beads for 60 s. The DNA was extracted in the small diameter tubing as described above. The extracted DNA was quantified by measuring its absorbance at 260 nm using a NanoDrop ND-1000 spectrophotometer and referencing a standard curve.

### Statistical Analysis

All statistical analyses were performed in SigmaPlot 11.0. Analysis of variance (ANOVA) was used to determine statistical significance for data containing 3 or more sample populations, and a T test used for data containing 2 sample populations. A p-value <0.05 was considered significant.

## Results

### DNA Extraction from Pooled Human Urine Samples

**Figure 2:**
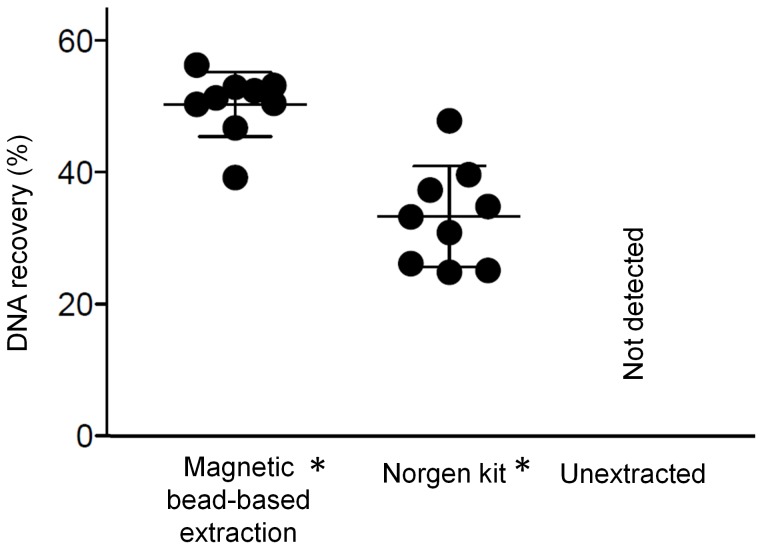
The DNA recovery for the magnetic bead-based extraction method and a commercially available laboratory-based kit are comparable. Though both are significantly higher than the unextracted sample, which is undetectable by PCR, there is no statistical difference between the two extraction methods (mean ± s.d., n = 9); * denotes statistically higher recovery than unextracted sample.

The magnetic bead-based extraction recovered 50±5% of TB IS6110 DNA spiked into 1 mL aliquots of pooled human urine (**Figure 2**, left). The commercially-available Norgen Biotek urine extraction kit recovered 33±6% of spiked DNA (**Figure 2**, middle). No spiked DNA was detectable in the unextracted urine sample (**Figure 2**, right) due to the presence of PCR inhibitors in urine. The primer efficiencies for samples extracted with the magnetic bead-based extraction and Norgen kit were 93% and 96%, respectively. Using the magnetic bead-based extraction, the DNA spiked into urine at an initial concentration of 5×10^5^ copies/µL was concentrated to 5×10^6^ copies/µL of water as determined by comparison to the PCR standard curve run at the same time.

### Effect of Urine Sample Volume on Final DNA Concentration in the Eluate

**Figure 3:**
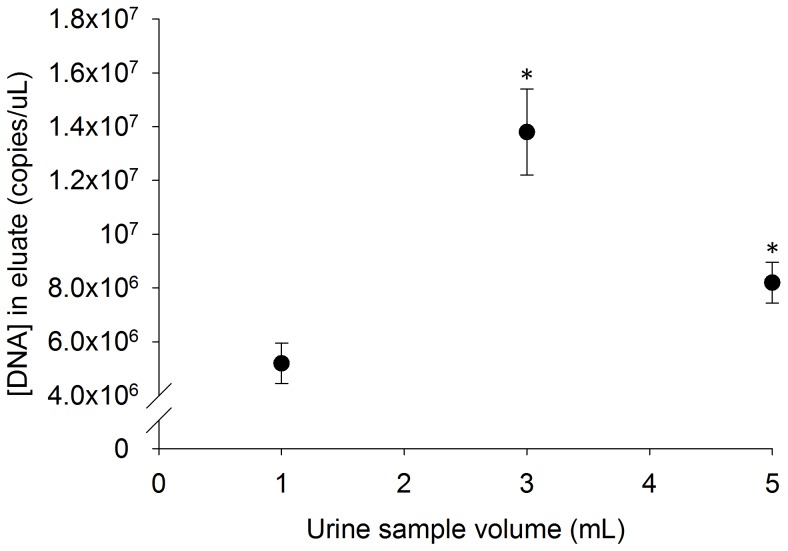
The final concentration of IS6110 DNA in the eluate following magnetic bead-based extraction of 3 and 5 mL spiked urine samples is significantly higher than the IS6110 DNA concentration in the eluate following the extraction of 1 mL samples (mean ± s.d., n = 3); * denotes statistically different from 1 mL sample.

As shown in **Figure 3**, the final concentration of spiked DNA in the eluate following extraction of the 1 mL urine sample was 5.2×10^6^±7.5×10^5^ copies/µL, corresponding to a recovery of 52%. The DNA concentration was significantly higher in both the 3 mL and 5 mL samples at 13.8×10^6^±16.0×10^5^ and 8.2×10^6^±7.6×10^5^ copies per µL. These corresponded to a total recovery of 46% of spiked DNA from the 3 mL sample and 17% from the 5 ml sample.

### Comparison of DNA Capture by Urine Collection Pipette and Freshly Prepared DNA-silica Adsorption Buffer

**Figure 4:**
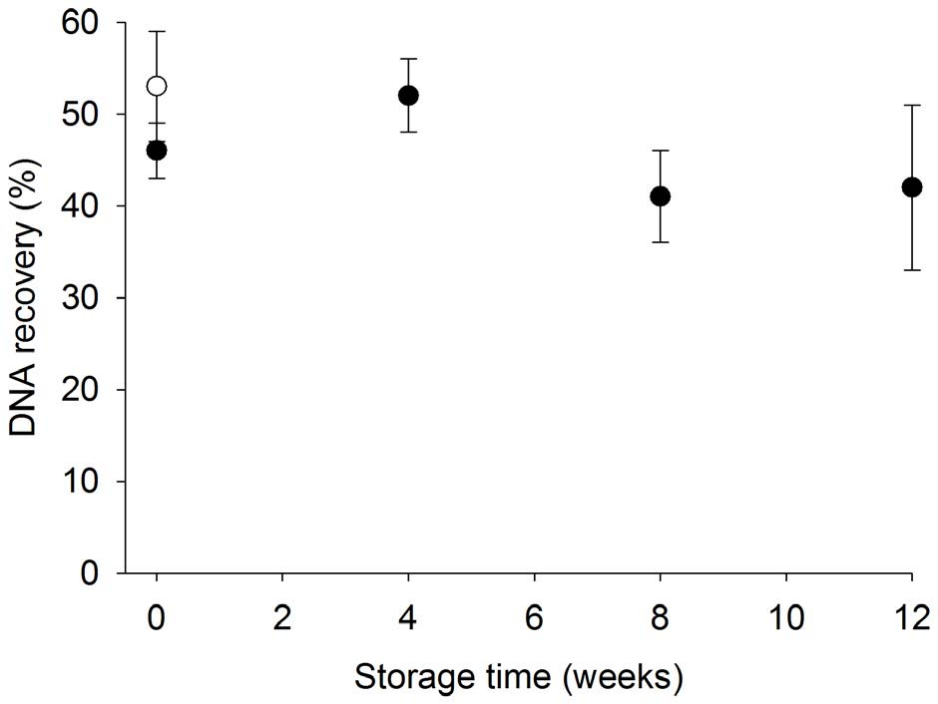
There is no significant difference in DNA recovery using the lyophilized urine collection pipette stored for 0, 4, 8, or 12 weeks (black circles) compared to freshly prepared adsorption reagents (white circle) (mean ± s.d., n = 3,).

There was no significant difference in recovery of DNA from the small diameter tubing following DNA capture in the urine collection pipette, which was stored in a lyophilized state, compared to a centrifuge tube containing freshly prepared adsorption reagents. The urine collection pipette method recovered 46±6%, 52±4%, 45±3%, and 42±9% of spiked DNA following urine collection pipette storage for 0, 4, 8, or 12 weeks, respectively, compared to 53±6% of spiked DNA using the freshly prepared DNA-silica adsorption buffer (**Figure 4**).

### Effect of Spiked DNA Concentration on DNA Recovery

**Figure 5:**
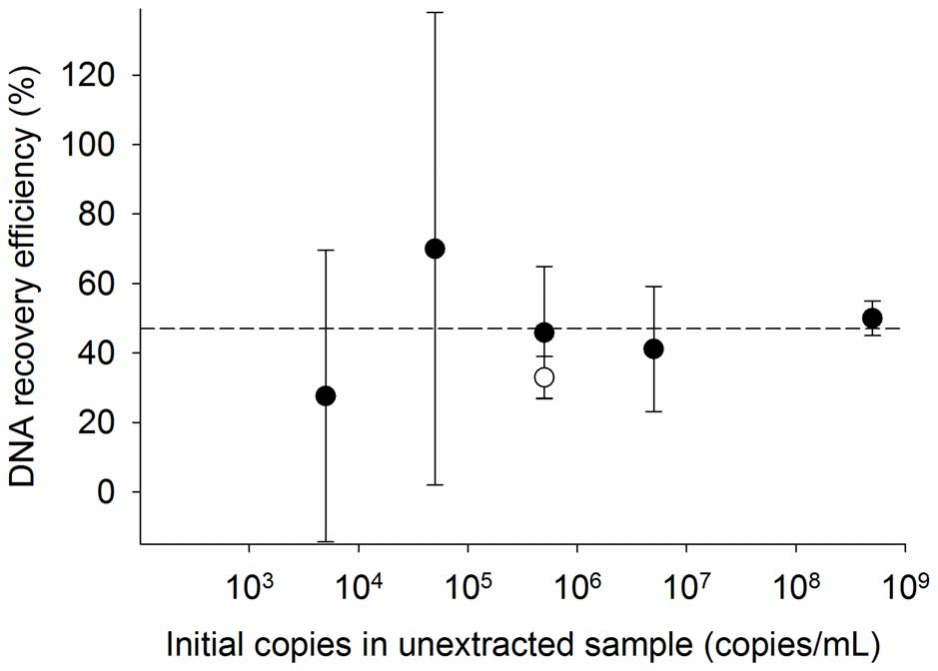
The recovery efficiency of DNA from human urine samples using magnetic bead-based extraction does not change as the number of copies spiked into the 1 mL human urine sample is increased (mean ± s.d., n = 3). The average DNA recovery efficiency of the magnetic bead-based extraction method is shown as a dotted line. The recovery efficiency of the Norgen kit is shown at an initial DNA concentration of 5×10^5^ copies/mL (white circle).

As the initial number of copies spiked into the 1 mL human urine sample was increased from 5×10^3^ to 5×10^8^, the percentage of DNA recovery from human urine samples did not change significantly over the range tested (**Figure 5**). DNA recovery was 28±42%, 70±68%, 46±19%, 41±18%, and 50±5% from urine spiked with 5×10^3^ copies DNA/mL, 5×10^4^ copies DNA/mL, 5×10^5^ copies DNA/mL, 5×10^6^ copies DNA/mL, and 5×10^8^ copies DNA/mL, respectively.

### PCR Limit of Detection

**Figure 6:**
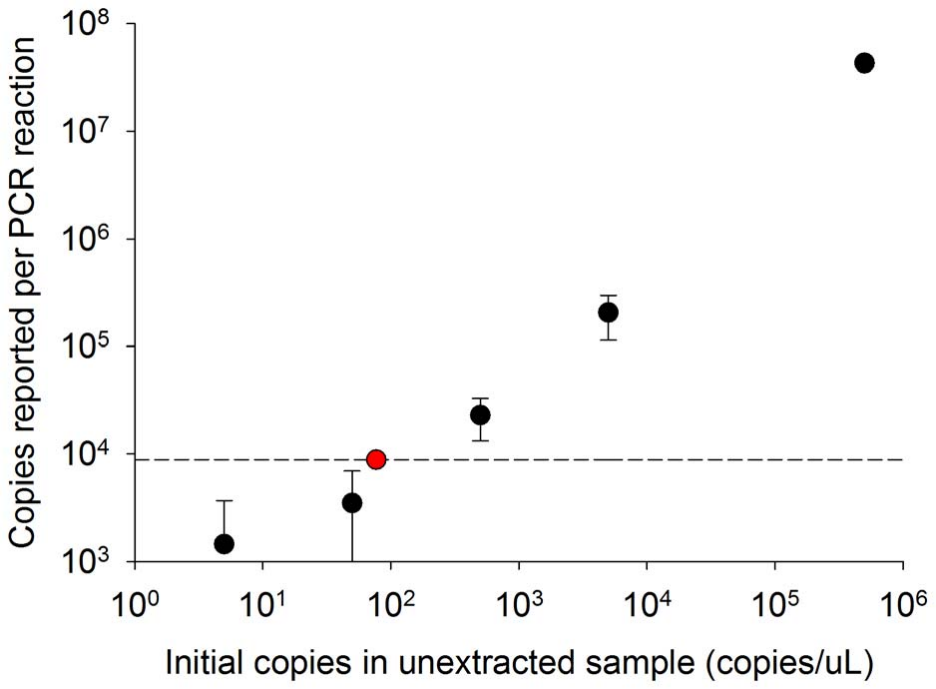
The LOD of the PCR reaction is shown at 8800 copies (dotted line). The lowest initial concentration of IS6110 DNA detectable at the LOD was determined to be 77 copies/µL at the point at which this intersects the PCR-concentration curve (red circle) (mean ± s.d., n = 3).

The PCR LOD is a reflection of background fluorescence due to primer dimers and other PCR artifacts. Following extraction of a 1 mL human urine sample using the magnetic bead-based method, fluorescence corresponding to 1300±2500 copies of DNA were reported by the PCR reaction. This resulted in a 3 s.d. PCR LOD of roughly 8800 copies (**Figure 6**, dotted line). It was determined that the concentration of IS6110 DNA in the initial human urine samples must be at least 77 copies/µL (**Figure 6**, red circle) to be detectable in the eluate at the calculated PCR LOD.

### Effect of DNA Length on DNA Adsorption to Silica

**Figure 7:**
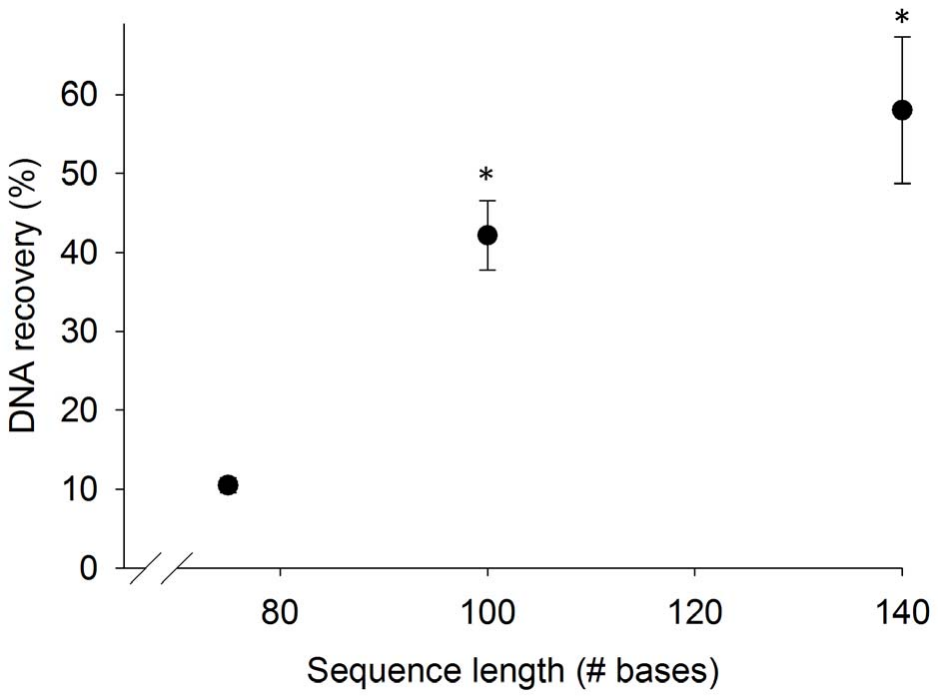
DNA recovery is significantly increased with increasing DNA sequence length (mean ± s.d., n = 3). * denotes statistically different than 75 bp sequence.

Increasing the length of target DNA increased the recovery of the magnetic bead-based extraction. As shown in **Figure 7**, when spiked with a 75 bp DNA sequence, the magnetic bead-based extraction recovered 10±0.9% of the sample from 1 mL of human urine. When the length of the DNA sequence was increased to 100 or 140 bases, the recovery increased to 42±4% and to 58±9%, respectively.

### Adsorption and Elution Kinetics of Silica-coated Magnetic Beads

**Figure 8:**
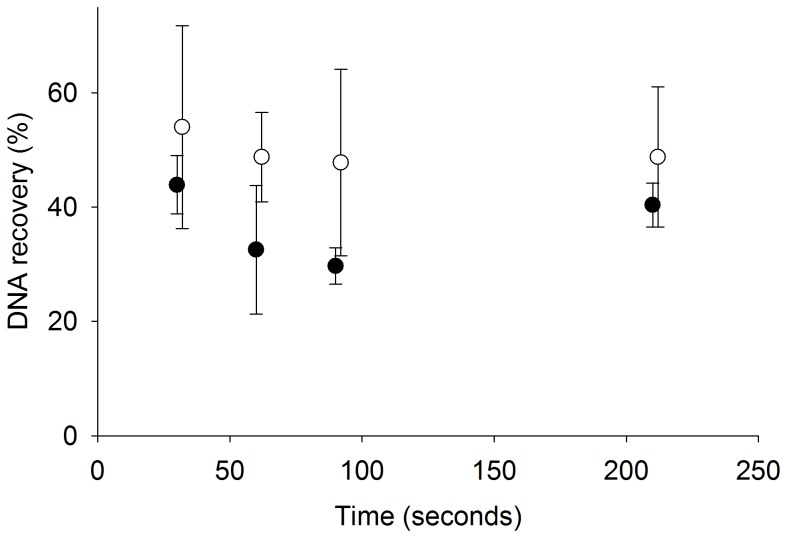
There was no significant change in DNA recovery with increased adsorption (white circles) or elution (black circles) times (mean ± s.d., n = 3). In each experiment, the adsorption time was held at 30 s while the elution time was varied (black circles), and the elution time was held at 30 s while the adsorption time was varied (white circles). To more clearly illustrate the error bars on each data point, the adsorption data was plotted two seconds to the right at each time point.

The percentage of DNA recovered from pooled human urine samples does not increase with increased adsorption or elution times. As shown in **Figure 8**, 54±18% is recovered from extractions with 30 s of adsorption time (white circles), and 44±5% of DNA is recovered from extractions with 30 s of elution time (black circles). As both adsorption and elution times are increased from 30 to 210 s there is no significant increase in the percentage of DNA recovered. In the ideal case, the black and white circles plotted at 30 s would be identical, because they have identical adsorption and elution times. The resulting minimal difference between the two data sets could simply be due to variation in PCR or the fact that the adsorption and elution data sets were collected on two separate days. As the error bars indicate, these points have a high degree of variation which masks any difference between them.

### Effect of Number of Magnetic Beads on DNA Recovery

**Figure 9:**
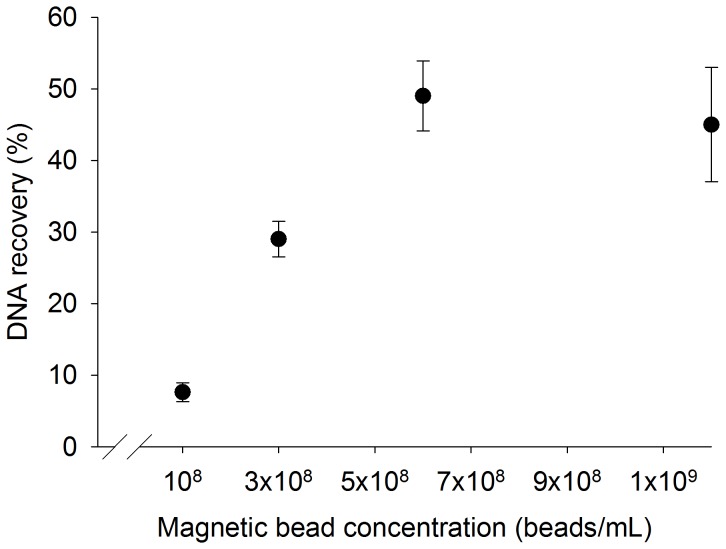
The percentage of DNA recovered from 1 mL urine samples increases as the concentration of magnetic beads increases to 6×10^8^ beads/mL (mean ± s.d., n = 3).

The percentage of DNA recovered from 1 mL urine samples increases as the number of magnetic beads is increased to 6×10^8^ beads/mL of urine. After this point, the recovery of DNA does not increase as additional beads are added. As shown in **Figure 9**, 7.6±1% of spiked DNA is recovered from extractions performed with 1×10^8^ beads/mL, 29±3% from extractions performed with 3×10^8^ beads/mL, 49±5% from extractions performed with 6×10^8^ beads/mL, and 45±8% from extractions performed with 12×10^8^ beads/mL.

### DNA Extraction Capacity of Silica-coated Magnetic Beads

**Figure 10:**
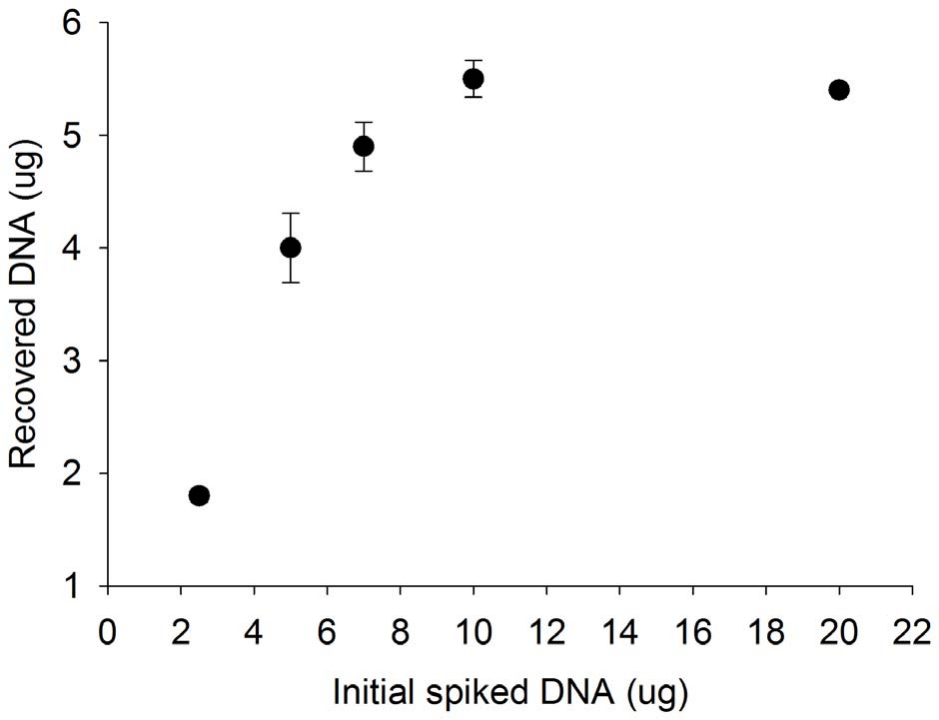
As the mass of spiked DNA is increased the mass of DNA recovered using the magnetic bead-based method increases and then plateaus at 5.5 µg (mean ± s.d., n = 3).

The maximum quantity of total DNA that can be extracted from TE buffer with 6×10^8^ magnetic beads is 5.5 µg, or ∼9 fg of DNA per bead. The mass of extracted DNA increases proportionally with an increase in magnetic beads and then plateaus. As shown in **Figure 10**, 1.8±0.1 µg of DNA was recovered when the extracted sample was spiked with 2.5 µg of DNA. The recovered DNA increased to 4.0±0.3 µg when the sample was spiked with 5 µg of DNA and 4.9±0.2 µg when spiked with 7 µg of DNA. As the quantity of spiked DNA was increased to 10 µg and subsequently to 20 µg, the quantity of extracted DNA was maximized at 5.5±0.2 µg and 5.4±0.1 µg, respectively. Based on the extraction capacity of the beads and an expectation of 250 ng DNA/mL of urine [24], this translates to a minimum of ∼2.7×10^7^ beads.

## Discussion

When biomarkers are present in low concentrations within patient samples, a large volume sample, such as urine, can maximize the number of biomarkers available for use in a molecular diagnostic test. However, processing large volumes is a challenge, as these large samples are difficult to interface with DNA extraction technologies such as microfluidic devices, particularly in a low resource setting. In our approach, magnetic beads were distributed in a large volume for nucleic acid adsorption and then transferred directly into small diameter tubing. Here, the beads were collected and the adsorbed nucleic acids concentrated in a format compatible with PCR. The magnetic bead-based method for extracting DNA from urine had an average recovery of 50% while concentrating the DNA ten-fold (**Figure 2**). The high primer efficiency for the magnetic bead-based extraction calculated from the PCR reaction (93%) indicated that the extracted DNA was sufficiently separated from PCR inhibitors found in urine samples. In optimization studies of the device, the elution volume was varied from 25 to 500 µL to determine its effect on DNA recovery. Although increasing the elution volume increased the total recovery of DNA from each sample, the end result was a more dilute final product. We chose a 50 µL elution volume because it yielded the highest final biomarker concentration.

The DNA biomarkers present in up to 5 mL of urine were captured onto the surface of silica-coated magnetic beads pre-lyophilized with the chaotropic salts necessary for nucleic acid-silica adsorption in the bulb of a 5 mL transfer pipette (**Figure 1A**). This design streamlines the concentration of DNA from large volume samples by eliminating the need for additional sample handling and pipetting steps that were required in our previous reports [21], [22]. Following adsorption of nucleic acids to the magnetic beads, the transfer pipette was used to directly introduce the sample into the small diameter tubing without additional pipettors or disposable supplies, an advantage in low resource settings. The transfer pipette bulb was large enough to contain the entire sample and allowed the beads to be easily distributed throughout the volume for maximum nucleic acid adsorption. For this study, the 1 mL sample containing the beads was introduced into the tubing resulting in a 60 cm length. However, this step is not necessary, and the overall tubing length can be shortened to ∼15 cm by disposing of the majority of the urine sample and transferring only the magnetic beads into the small diameter tubing.

The lyophilized urine collection pipette appears to have some long-term storage advantages, since lyophilized and freshly prepared reagents within the transfer pipette produce similar results (**Figure 4**). This is partially due to the fact that the adsorption buffer consists only of salt and magnetic beads, which would be expected to have a long shelf-life in an unhydrated state but are easily resuspended. The transfer pipettes were prepared in batches of 50 and stored at room temperature for up to 12 weeks prior to use. Over the 12 week storage period, there was no reduction in device performance (**Figure 4**). The benefits of long term storage may be advantageous in low resource settings where reliable temperature control is not readily available. Furthermore, the composition of the lyophilized contents of the transfer pipette can be modified to target other biomarkers in additional sample matrices such as blood and saliva.

Although 1 mL urine samples were used for the majority of this study, the capacity of the urine collection pipette allows a maximum of 5 mL of urine to be processed with the magnetic bead-based method. This volume is ten-fold greater than the maximum volume that can be processed using a typical microfluidic extraction device. As shown in **Figure 3**, processing larger volumes of urine containing the same starting concentration of spiked DNA as the 1 mL urine sample yields significantly higher DNA concentrations in the eluate, thus improving the likelihood of detecting any biomarkers of interest. This is especially beneficial in cases, such as Tr-DNA, where the expected concentration of biomarkers has not been well characterized but is thought to be low. Since urine can be easily obtained in these volumes, it would be possible to improve detection simply by increasing the sample volume used.

There is a decrease in DNA concentration in the eluate of the 5 mL urine sample compared to the 3 mL urine sample. Because the binding capacity of the beads limits the number of biomarkers that can be captured within a given sample, it is important to consider this when increasing the urine sample volume. The maximum mass of DNA that can be extracted with the magnetic beads was found to be 5.5 µg DNA/6×10^8^ beads (**Figure 10**). This is ∼20 times more DNA than the 250 ng expected in a 1 mL urine sample, or ∼4 times more than the 1.25 µg expected in a 5 mL urine sample [24].

Although there is ample DNA capacity on the beads for a pure DNA sample containing the equivalent amount of DNA as a 5 mL urine sample, the additional PCR inhibitors present in the larger urine sample could be one cause of the decreased concentration. However, the scalability of the magnetic bead-based extraction would allow us to optimize for a 5 mL sample to take advantage of the increased biomarkers in a larger sample. As shown in **Figure 9**, increasing the number of magnetic beads increases DNA recovery up to a point. Additionally, increasing the volumes of wash solutions to remove additional inhibitors may further improve the final DNA concentration in larger samples. With further optimization and a higher capacity transfer pipette, the magnetic bead-based method could be adapted to extract biomarkers from increasingly large urine volumes as high as 20 mL. This drastic increase in volume may require a longer adsorption step, but the opportunity to capture more biomarkers may be worth the trade-off in total processing time.

Two factors limit the general utility of this approach for detection of disease biomarkers excreted in urine. The first is the availability of biomarkers in urine. Relatively little is known about DNA excreted in urine, but there is evidence that a nucleic acid background of 40–250 ng/mL is normally present [24]. Using gel electrophoresis to visualize Tr-DNA extracted from human urine provided by healthy volunteers, Melkonyan et. al determined that the expected size of these DNA fragments in urine is less than 250 bp, with the majority of the fragments being between 10 and 150 bp [4]. With respect to diagnosis of TB, the use of biomarkers in urine is controversial. Though some studies have successfully detected TB DNA in urine [6], [7], others have achieved poor results [5], [25], [26]. This may be due to lack of standardization in sample collection and processing, secondary infections, medication, or differences in patient populations. Active TB infection is the most likely disease state for detection, since in latent TB the bacteria are sequestered within the lungs [27], and one would expect much lower levels of TB DNA biomarker to be available in the blood and urine. Perhaps the biomarker recovery strategy described here will enable more definitive studies of Tr-DNA as a potential TB diagnostic. Additionally, there are other potential applications for which our technology could be useful, including the extraction of carbohydrates for the diagnosis of TB using LAM, or the extraction of nucleic acid biomarkers for chlamydia, gonorrhea, and prostate cancer [28]–[30].

DNA segment length affects the binding properties of the silica-coated beads and is a second factor limiting the diagnostic utility of this approach. We found that sequences as short as 75 bp can be extracted from human urine following adsorption in the transfer pipette bulb, and that the recovery of DNA increases as the sequence length is increased. These results agree with previously reported data that suggest the minimum nucleic acid length that can be recovered using silica adsorption is 60 bp, and that the recovery of nucleic acid is lower near this limit [31]. As demonstrated in **Figure 7**, the recovery of 100 and 140 bp DNA sequences is increased approximately four-fold and six-fold, respectively, compared to a 75 bp sequence. Although the recovery of DNA decreases as the sequence length decreases, the longer sequences tested fall within the expected size range for Tr-DNA as reported by Melkonyan et. al [4].

In patient samples exhibiting expected patient variability, the relationship between TB Tr-DNA measurements and clinical diagnosis remains to be determined. In this study, one large stock of pooled human urine samples was used in an effort to minimize sample-to-sample variability of PCR inhibitors and account for the expected difference in nucleic acid concentration between patient samples. A single concentration of synthetic TB IS6110 biomarker was spiked into each sample to create a controlled surrogate patient sample for the validation of our device. Using this approach, we found that DNA recovery from human urine samples remained constant as the number of copies of spiked DNA increased (**Figure 5**). This is an advantage, because when biomarkers are present in low concentrations, the magnetic bead-based method can still be used to effectively extract a high percentage of the biomarkers. As the concentration of spiked DNA was increased, the sample variance decreased. This was an expected trend since at low copy numbers the signal is more difficult to distinguish from noise, a characteristic not only of the extraction process, but also of the PCR.

After determining the LOD of the PCR reaction used in this study, we found that 77 copies/µL was the minimum concentration of IS6110 DNA that must be present in a 1 mL human urine sample to be detectable by PCR following extraction with the magnetic bead-based method (**Figure 6** **)**. This reported value is a function of the PCR protocol, the sample preparation method, and the limited sample-to-sample variability of PCR inhibitors present in the pooled human urine samples. There is currently no clinical data available to determine the expected initial human urine biomarker concentration that would be necessary to utilize Tr-DNA samples for TB diagnosis. We expect that if the use of Tr-DNA for clinical diagnosis becomes standardized in the future, our reported biomarker concentration will be useful in this field of study. We also anticipate that the minimum required biomarker concentration would be different for alternative downstream detection strategies.

One of the attractive features of the magnetic bead-based extraction strategy is that it is amenable to low resource settings. First, urine samples are easy to obtain noninvasively and can usually be obtained in much higher volumes than other samples. The use of urine also circumvents the cultural taboos and cross contamination concerns with using blood samples. Second, because of the simplicity and storage capabilities of the urine collection pipette, the magnetic bead-based extraction does not require refrigeration or special handling, both of which are highly desirable in a low resource setting. Third, the reagent costs are relatively low and are estimated to be less than $1 per extraction. Fourth, a short processing time is essential in low resource settings where patients often require an immediate diagnosis. The rapid DNA adsorption and elution from the magnetic beads minimizes the processing time required to achieve maximum DNA recovery (**Figure 8**), thus reducing the overall time-to-diagnosis. The magnetic bead-based extraction recovers comparable DNA to the Norgen kit and can be completed nearly 3 times faster. Finally, although PCR was used as the nucleic acid detection method to validate the work done in this study, the extracted nucleic acids are expected to be compatible with a variety of downstream detection strategies that may be better suited for use in low resource settings, such as isothermal PCR. One of the long-term goals of this research is to incorporate an isothermal PCR amplification and detection scheme directly into the small diameter tubing to identify biomarkers-of-interest after extraction.

Because there is no comparable low resource urine extraction technology, we chose to validate our magnetic bead-based extraction by comparing its performance to the commercially available laboratory-based Norgen urine extraction kit. Though it performs well, the Norgen kit requires multiple pipetting and centrifugation steps which make it unsuitable for use in a low resource setting. The magnetic bead-based extraction presented here performs similar to or better than the Norgen kit. However, in the field, the magnetic bead-based extraction would be available as a preloaded cassette containing all necessary processing solutions sealed within the tubing. For each extraction, processing would require only a sample collection step within the urine collection pipette followed by the transfer of magnetic beads down the length of the small diameter tubing using an external magnet.

The magnetic bead-based extraction method effectively captures nucleic acids from large volume urine samples and concentrates them for downstream detection. The urine collection pipette in combination with the pre-arrayed solutions in the small diameter tubing achieves high nucleic acid recovery from easily obtainable clinical human urine samples. This approach, therefore, has potential utility for urine-based diagnosis of active TB in low resource settings and other diagnostic applications found to be associated with 60–250 bp DNA fragments excreted in urine.
